# Intravenous immunoglobulin for the treatment of autoimmune encephalopathy in children with autism

**DOI:** 10.1038/s41398-018-0214-7

**Published:** 2018-08-10

**Authors:** Kathleen Connery, Marie Tippett, Leanna M. Delhey, Shannon Rose, John C. Slattery, Stephen G. Kahler, Juergen Hahn, Uwe Kruger, Madeleine W. Cunningham, Craig Shimasaki, Richard E. Frye

**Affiliations:** 1 University of Arkansas for Medical Sciences, Little Rock, AR 77205 USA; 2 BioROSA Technologies Inc, San Francisco, CA 94103 USA; 3 Department of Biomedical Engineering, Rensselaer Polytechnic Institute, Troy, NY 12180 USA; 4 Center for Biotechnology & Interdisciplinary Studies, Rensselaer Polytechnic Institute, Troy, NY 12180 USA; 5 Department of Microbiology and Immunology, University of Oklahoma Health Sciences Center, Oklahoma City, OK 73104 USA; 6 Moleculera Labs, Inc, Oklahoma City, OK USA; 7 Barrow Neurological Institute at Phoenix Children’s Hospital, Phoenix, AZ USA; 8 Department of Child Health, University of Arizona College of Medicine - Phoenix, Phoenix, AZ USA

## Abstract

The identification of brain-targeted autoantibodies in children with autism spectrum disorder (ASD) raises the possibility of autoimmune encephalopathy (AIE). Intravenous immunoglobulin (IVIG) is effective for AIE and for some children with ASD. Here, we present the largest case series of children with ASD treated with IVIG. Through an ASD clinic, we screened 82 children for AIE, 80 of them with ASD. IVIG was recommended for 49 (60%) with 31 (38%) receiving the treatment under our care team. The majority of parents (90%) reported some improvement with 71% reporting improvements in two or more symptoms. In a subset of patients, Aberrant Behavior Checklist (ABC) and/or Social Responsiveness Scale (SRS) were completed before and during IVIG treatment. Statistically significant improvement occurred in the SRS and ABC. The antidopamine D2L receptor antibody, the anti-tubulin antibody and the ratio of the antidopamine D2L to D1 receptor antibodies were related to changes in the ABC. The Cunningham Panel predicted SRS, ABC, parent-based treatment responses with good accuracy. Adverse effects were common (62%) but mostly limited to the infusion period. Only two (6%) patients discontinued IVIG because of adverse effects. Overall, our open-label case series provides support for the possibility that some children with ASD may benefit from IVIG. Given that adverse effects are not uncommon, IVIG treatment needs to be considered cautiously. We identified immune biomarkers in select IVIG responders but larger cohorts are needed to study immune biomarkers in more detail. Our small open-label exploratory trial provides evidence supporting a neuroimmune subgroup in patients with ASD.

## Background

Autism spectrum disorder (ASD) is a behaviorally defined disorder, which now affects ~ 2% of children in the United States^1^. Although the standard-of-care for ASD is behavioral therapy, such therapy requires full-time engagement with one or several therapists for many years. In many cases, outcomes are suboptimal and/or incomplete^2^. Thus, medical therapies that can augment behavioral therapy are urgently needed.

Recent studies suggest that ASD is associated with a variety of physiological abnormalities including immune system dysfunction^3^. For example, a maternal immune response induced during gestation results in ASD-like behavior in offspring in the maternal immune activation (MIA) rodent^4,5^ and replacing the immune system using bone marrow transplant corrected many symptoms in a mouse model of Rett syndrome, a syndrome closely aligned with ASD^6^. In individuals with ASD, inflammatory cytokines are elevated in the blood^7^ and brain^8^ and microglia are active in the brain^8^. Children with ASD have autoantibodies to brain tissue such as myelin basic protein, serotonin receptors, brain endothelium, cerebellar tissue, and glutamic acid decarboxylase (GAD) as well as to non-brain tissue such as the folate receptor alpha (FRα)^9^ and mitochondria^3^. Children with autoantibodies are reported to have a more severe form of ASD^10–15^. The role of autoantibodies during gestation is exemplified by maternal antibodies to fetal brain that have high specificity for the development of ASD in the offspring^16^ with a relatively more severe phenotype^17^ and brain enlargement^18^.

The overlap between ASD and Pediatric Autoimmune Neuropsychiatric Disorders Associated with Streptococcal Infection (PANDAS) and Pediatric Acute-Onset Neuropsychiatric Syndrome (PANS) has been undergoing study by Cunningham et al. (in preparation) over the last several years. PANDAS/PANS-associated autoantibodies accelerate the function of the calcium calmodulin-dependent protein kinase II (CaMKII) enzyme, a multifunctional enzyme highly concentrated in the brain that controls neurotransmission and neuronal excitability^19^ and regulates catecholamine^20^ and glutamate^21^ neurotransmission. Abnormalities in CaMKII function has been linked to movement and neuropsychiatric disorders in children^22,23^ and genetic abnormalities in the CaMKII genes are linked to intellectual disability^24^. Furthermore, previous studies have linked PANDAS/PANS-associated biomarkers with behaviors commonly seen in ASD. For example, high levels of either the anti-lysoganglioside GM1 antibody or CaMKII are associated with tics and obsessive-compulsive disorder behavior^25^ and high antidopamine D2L to D1 antibody ratio is associated with irritability, a frequently targeted ASD symptom^26^.

Brain-targeted autoantibodies in an individual with central nervous system dysfunction point toward a diagnosis of an autoimmune encephalopathy (AIE). In children and adolescents AIE is most commonly diagnosed as non-neoplastic limbic encephalitis, resulting from GAD^27–33^, voltage-gated potassium channel (VGKC)^29,34^, or *N*-methyl-d-aspartate (NMDA) receptor^34–38^ autoantibodies or without autoantibodies^28–30,34,39^. Seizure is the most common presenting symptom but children commonly also manifest motor, cognitive, behavioral, and/or psychiatric symptoms including aphasia^35^, declining academic functioning^27^, depression^29^, anxiety^29^, amnesia^29,34,35^, learning problems^29^, loss of social skills^38^, hallucinations^34^, agitation^34,39^, mood disorder^34^, behavioral changes^30,35,39^, decreased appetite^36^, irritability^36^, dysautonomia^30,34,39^, movement disorder^35^, peripheral neuropathy^32^, dystonia^33^, and insomnia^36,39^. Both GAD65^40^ and NMDA receptor autoantibodies^36–38^ have been associated with ASD with the NMDA receptor autoantibody being associated with regressive^36,38^ and late-onset^37^ ASD.

Case series^28–30,34,35^ and case reports^27,31–33,36,39^ confirm that Intravenous immunoglobulin (IVIG) is useful for AIE in children and adolescents. IVIG has been shown to improve symptoms of ASD, including aberrant behavior^41^, speech and social interaction^42^, and ASD-related behaviors^43^. A small double-blind placebo-controlled study reported significant improvements in children with ASD receiving IVIG on the Aberrant Behavior Checklist (ABC) compared with those receiving placebo^44^. Another study demonstrated neurodevelopmental regression when discontinuing IVIG treatment^41^. In contrast, smaller case series did not find improvements in children with ASD with IVIG treatment^45,46^. As IVIG has been used to treat PANDAS/PANS symptoms in a placebo-controlled study^47^, case reports^48^, and case series^49^, IVIG could be a treatment for children with ASD with PANDAS/PANS symptoms.

Given that children with ASD may manifest brain autoantibodies, we screened patients in an ASD clinic who did not respond to standard interventions for AIE. Selected patients with possible AIE were offered IVIG as a potential treatment. As part of our clinical protocol, families were asked to complete specific behavioral questionnaires, allowing the examination of the change in behavioral ratings for patients that received IVIG. In addition, clinical notes were analyzed to determine symptoms improvement, adverse effect, and treatment course. We also examined if immune biomarkers could predict response to IVIG treatment. Lastly, we provide several cases as illustrations of the treatment course with IVIG. This report provides insight to the possible utility of IVIG treatment in children with ASD as well as the potential limitations of this treatment.

## Methods

The Institutional Review Board at the University of Arkansas for Medical Sciences (Little Rock, AR) approved the study. Parents of participants provided written informed consent. The study is registered as NCT02003170 on clinicaltrials.gov.

All patients evaluated in our ASD multispecialty clinic for AIE were included in this study. The majority of the patients (80/82) were diagnosis with ASD, except for two females, one who was a sibling of a male with ASD and AIE who was treated for severe atypical learning disabilities and attention deficit and one with severe tics and behavioral dysregulation.

ASD was diagnosed based upon one of the following criteria: (i) a gold-standard diagnostic instrument such as the Autism Diagnostic Observation Schedule and/or Autism Diagnostic Interview-Revised; (ii) the state of Arkansas diagnostic standard, defined as agreement of a physician, psychologist and speech therapist; and/or (iii) Diagnostic and Statistical Manual of Mental Disorders (DSM) diagnosis by a physician along with standardized validated questionnaires and diagnosis confirmation by the Principal Investigator (R.E.F.). We have validated that this criteria captures an accurate diagnosis of ASD in our previous studies by re-evaluating a portion of the participants with the ADI-R^50–53^.

Table S1 provides participant characteristics. Comorbid conditions were derived from a parent-reported medical questionnaire and from review of medical records. Regression (defined as loss of already obtained skills) was defined in detail in our questionnaire. Questions regarding regression included the timing, specific skills lost, duration of the regression, trigger (if known), and whether or not there were multiple regressions or a single regression. This method for assessing medical comorbidities has been used in several of our previous studies^50–53^.

### Immune markers measured

The evaluation for AIE focused on detecting brain autoantibodies as measured by several panels. Paraneoplastic panel (PNP; Mayo Medical Laboratories, Rochester, MN) measures antineuronal nuclear, anti-glial nuclear, Purkinje cytoplasmic, P/Q and N-type calcium, and VGKC antibodies as well as GAD65, AMPA, NMDA, and GABA-B receptor antibodies. Because GAD65^40^ and NMDA receptor^36–38,54^ autoantibodies have been associated with ASD, these two autoantibodies were measured specifically in most patients. The Cunningham panel (antidopamine D1 receptor (D1R), antidopamine D2L receptor (D2R), anti-lysoganglioside GM1, anti-tubulin, CaMKII; Moleculera Lab Inc, Oklahoma City, OK) was also performed as the symptoms associated with these autoantibodies overlap with behavioral symptoms of ASD. In some patients, the FRα autoantibody was measured because of its close association with ASD^9,51,52,55^. Some patients also had tests performed for markers of immune activation such as C3, C4, CH50, C-reactive protein, and erythrocyte sedimentation rate. Using findings from these laboratory tests along with clinical symptoms, the need for treatment of AIE with IVIG was considered, which is the focus of this report.

### Direct enzyme-linked immunosorbent assay (ELISA)

Ninety-six-well microtiter plates (Greiner Bio-One, Monroe, NC) were coated with 50 μL of antigen in 100 mM carbonate/bicarbonate buffer (pH 9.6) and stored up to 2 weeks at 4 °C. Antigen coating concentrations were as follows: 10 µg/mL of purified tubulin (MP Biomedicals, Santa Ana, CA), 10 µg/mL of dopamine D1 receptor (D1R, Perkin Elmer, Waltham, MA), 10 µg/mL dopamine D2L receptor (D2R, Perkin Elmer), and 20 µg/mL of lysoganglioside G_M1_ (Sigma Aldrich, Darmstadt, Germany). Tubulin-, D1R-, and D2R-coated plates were washed three times with phosphate-buffered saline (PBS, pH 7.2) containing 0.1% Tween (ThermoFisher Scientific, Waltham, MA). Lysoganglioside-coated plates were washed three times in PBS without Tween in all steps. Plates were blocked with 1% bovine serum albumin (ThermoFisher Scientific) in PBS for 60 minutes at 37 °C. Serum or cerebrospinal fluid (CSF) samples serially diluted in 1% BSA (in PBS) were added to washed plates, then incubated overnight at 4 °C. The next day, plates were washed as described above and primary IgG antibody binding was detected by adding 50 µL per well of diluted alkaline phosphatase-conjugated goat anti-human ƴ-chain-specific secondary antibody (polyclonal, Cat# A3312, Sigma Aldrich) and incubated for 60 min at 37 °C. Final dilution of secondary antibody was determined empirically for each antigen and validated for every new antibody lot. Plates were developed at 26 °C for 2 h ± 15 min with 50 µL per well of 1 mg/mL *p*-nitrophenylphosphatase (Sigma Aldrich) in 0.1 M diethanolamine buffer (pH 9.8). Optical density values were measured at 405 nm on an automated BioTek microplate reader (BioTek Instruments, Winooski, VT) and corrected by blanks (wells coated with antigen, without serum added). All samples were assayed in duplicate and averaged. Duplicates not matching with < 20% variance were repeated. Titers represent the serum dilution at optical density of 0.1 at 405 nm after 2 h. Known positive and negative control samples were included on each plate to standardize and monitor assay performance^20,29^. Each new lot of all reagents and antibodies were validated using serum samples with known titers.

### Cell culture

SK-N-SH human neuroblastoma cells obtained from American Type Culture Collection (HTB-11, Manassas, VA) were grown in complete F12-Dulbecco’s Modified Eagle Medium (ThermoFisher Scientific) as previously described^15^. Complete media contained 10% fetal bovine serum (ThermoFisher Scientific) and 1% penicillin–streptomycin antibiotic (ThermoFisher Scientific). Cellular extracts used in the CaMKII assay were centrifuged at 15,000 rpm for 10 min at 4 °C. Protein concentrations of the extracts were determined by Bradford assay using the Protein Assay Kit II (Bio-Rad, Hercules, CA).

### CaMKII activity assay

Assay for CaMKII activity was performed as previously described^15^. In brief, SK-N-SH cells were plated in six-well plates at 2.5 million cells/well and incubated overnight in complete F12-DMEM, at 37 °C with 5% CO2. The next day, cells were serum starved for 30 minutes in serum-free F12 media with 2 mM CaCl2, 2 mM KCl, and 0.4 mM MgCl2, then stimulated for 30 minutes with patient sera or CSF diluted 1:100 in the same media or with media alone (basal control). Cells were harvested, centrifuged, solubilized in 0.165 mL of protein extraction buffer with protease inhibitors (Soybean Trypsin Inhibitor, PMSF, Leupeptin, and Aprotinin, Sigma Aldrich, St. Louis, MO), and homogenized. Enzymatic activity was measured using the CaMKII assay system (Promega, Madison, WI) per manufacturer’s instructions. In brief, 5 μL of cell lysate was incubated with 50 μM peptide substrate, buffers and ATP [γ-^32^P] (Perkin Elmer) for 2 minutes at 30 °C. Samples were spotted onto capture membranes and washed. Radioactivity retained on the membrane was measured by scintillation counter (Beckman Coulter, Indianapolis, IN) and used to calculate specific activity of the CaMKII enzyme (pmoL/min/μg) as described in kit instructions. The protein concentration of each sample was used to standardize the CaMKII enzyme activity, and the percentage of specific activity of baseline (basal control) was calculated for each sample where the basal level was set at 100%. All samples were assayed in triplicate and results were averaged. Sera from patients with known high and low CaMKII activity and a basal control sample were included to standardize the assay.

### Parent-reported clinical outcomes

The first author, who was not involved in patient care, performed a qualitative–quantitative analysis of clinical notes. The senior author (R.E.F.), the primary treating physician, confirmed this analysis for accuracy. The notes were analyzed for specific symptoms mentioned in regards to improvements and worsening. Areas of improvement were quantified if they occurred in ≥ 10% of the participants. Symptom improvement was classified into eight non-exclusive categories (See Table 2), “other” and “no improvement.” The frequency of these reported improvements across patients was calculated and the total number of improved symptoms was also analyzed for each patient. A similar chart review was performed for determining frequency of adverse effects, although there was no lower limit for reporting the frequency of adverse effects.

### Cognitive and behavioral outcome measures

The ABC is a 58-item questionnaire^56^ that measures disruptive behaviors and has convergent and divergent validity^57^. The Social Responsiveness Scale (SRS) is a 65-item questionnaire that measures the severity of social skill deficits across five domains^58^, which has good concordance with the ADOS^59^. Caretakers are asked to complete these questionnaires prior to each clinical visit to assist in the evaluation of the change in symptoms at each visit. One important caveat is that caretakers were not required to fill out these questionnaires as declining did not ethically prevent their child from appropriate medical care.

### Statistical analysis

Statistical analyses were performed with SAS 9.4 (SAS Institute Inc., Cary, NC) and MATLAB (The Mathworks, Natick, MA). Graphs were produced using Excel version 14.0 (Microsoft Corp, Redmond, WA). To determine whether any significant change occurred in behavior questionnaires with treatment an analysis of variance implemented as a mixed-model regression was utilized. The models included random effect of time (before/during treatment) and intercept to account for individual symptom level. The models tested the a priori hypothesis that a significant change in the outcome measure occurred with treatment and used a *α* ≤ 0.05. All of the available questionnaire data were used for each participant. There was no imputation for missing data. Participants without questionnaire data both before and after the start of treatment were excluded. The Cohen’s *d’* effect size was calculated for each statistical comparison.

We sought to evaluate whether biomarkers of autoimmunity could predict treatment response. As the Cunningham panel was the only frequently abnormal panel, only the Cunningham panel was used to predict treatment response. We examined the relationship between the change in questionnaire score with IVIG treatment and the baseline components of the Cunningham panel. Only the total scores for the ABC and SRS questionnaires were examined to reduce excessive statistical comparisons. However, we also examined the relationship between the Anti-Dopamine D2L/D1 titer ratio and irritability subscale of the ABC, as this relationship was previously reported^26^. We applied a logarithmic transformation with a base of 2 to the autoantibody titers as they increase in exponential steps. As many individuals completed multiple questionnaires both before and during the treatment period, we calculated the average total score of all questionnaires during treatment minus the average total score prior to treatment for each individual as a difference score. We then calculated the correlation between this difference score vs immune biomarkers.

We then defined responders by examining the histogram of the change in the ABC and SRS scores to determine the distribution of responders. As the scale of the Cunningham panel components are quite different, in order to conduct calculations on the same scale across all components, as is standard is statistical linear models, were mean-centered at zero and normalized to have a standard deviation of unity. We then determined whether the components of the Cunningham panel could be used to predict which participants were responders and which where non-responders using Fisher Discriminant Analysis in MATLAB using in-house developed routine. Discriminant analysis creates a linear function that uses the input component (i.e., components of the Cunningham panel) to calculate a score that represent whether a particular participant is more like a responder or non-responses. The statistical distribution of these Fisher Discriminant Analysis scores was estimated via Kernel Density Estimation and a threshold was determined which could optimally divide the scores calculated by the linear discriminant function for the participants into responders and non-responders (i.e., a binary classifier). We report the accuracy, sensitivity, and specificity of the discriminant function.

## Results

### Characterization of AIE

NMDA receptor autoantibodies were negative in all of the 34 patients in which it was tested. GAD65 titer elevation was found in three (5%; 13.00, 0.11, 0.07;  normal (nl) < 0.02) of the 60 patients in whom it was tested. The PNP was positive for four (6%) of the 63 patients in which it was tested. For the PNP, two of the cases had VGCC autoantibodies (N-type 0.13; nl < 0.03; P/Q-type 0.05, nl < 0.02) and two had striated muscle autoantibodies (1:960 and 1:1920, nl < 1:120).

The Cunningham panel was considered to be is positive only when one of the four autoantibody titers was positive in the ELISA AND at the same time autoantibody-mediated CaMKII activation was positive (> 130 above the basal rate of CaMKII in SKNSH human neuronal cell lines as previously described^22^). In this way, the CaMKII elevation was used as a functional confirmation of the consequence of elevated autoantibodies in the ELISA. In the ELISA, the antineuronal autoantibodies were positive when anti-tubulin was ≥ 2000 titer, anti-lysoganglioside was ≥ 640 titer, anti-D1R was ≥ 4000 titer, and anti-D2R was ≥ 16,000 titer. Endpoint titers were determined at 0.092 cutoff and the titers were determined by routine ELISA as described above.

Using our criterion, the Cunningham panel was positive in 44 (57%) of the 77 patients in which it was performed. In 67% of the 33 cases, the Cunningham panel was negative because of a normal CaMKII, whereas it was negative because of normal ELISA autoantibody titers in 30% of the cases and was normal for both ELISA autoantibodies and CaMKII in only one case.

In addition to the aforementioned tests, patients with severe behavioral or medical symptoms including drug-resistance epilepsy and/or immunodeficiency were considered candidates for IVIG treatment. If the patient was previously non-responsive to IVIG treatment, alternative treatments were considered. Also, consideration was given to whether other treatments should be tried as an alternative to IVIG. The severity of the ASD and associated behavioral and other symptoms were a determinant when deciding whether a trial of IVIG was warranted.

**Fig. 1:**
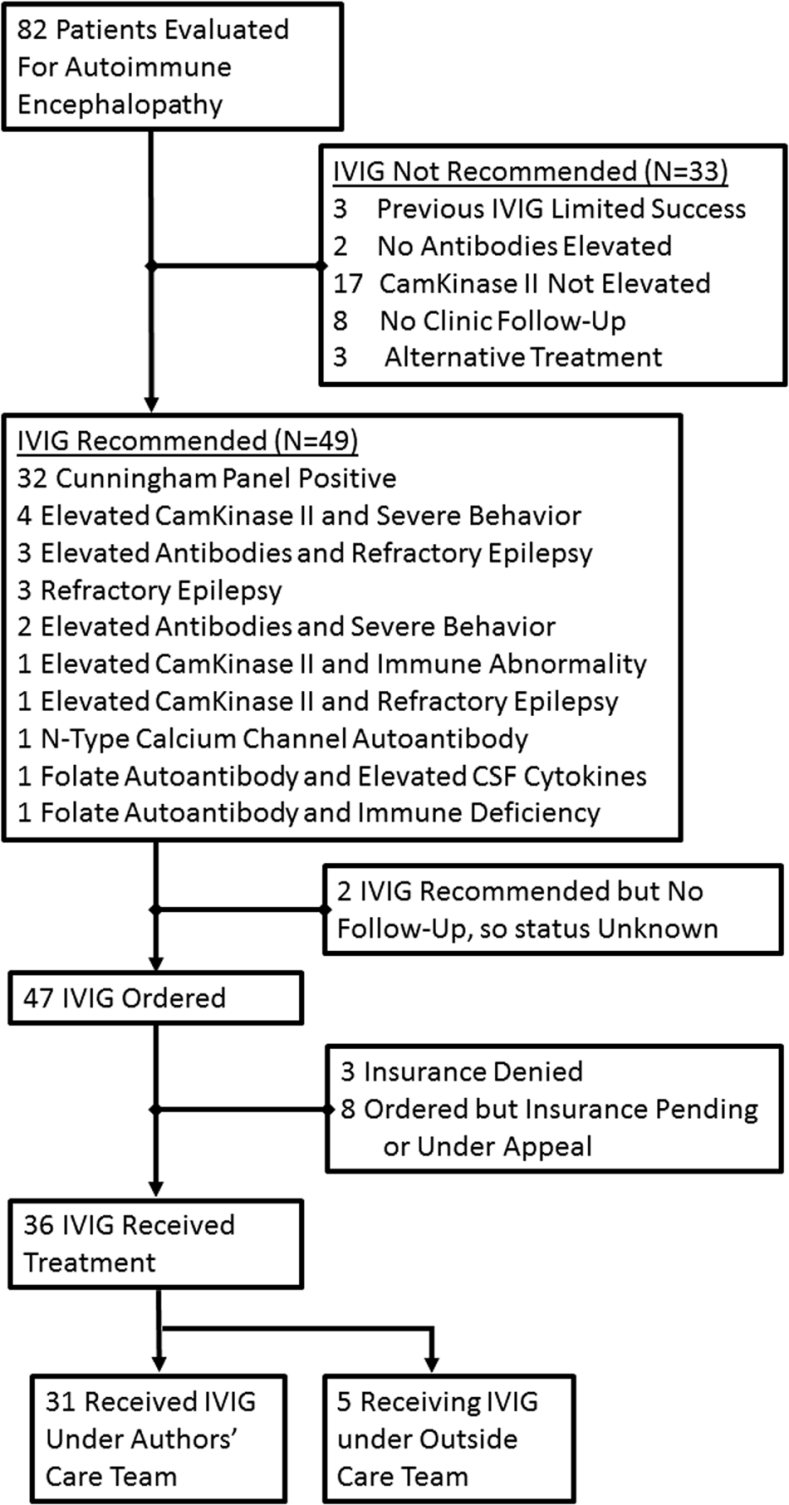
Flowchart for patient evaluation, criteria for diagnosis with autoimmune encephalopathy and recommended treatment

A trial of IVIG was recommended in 49 (60%) of the patients evaluated (See Fig. 1). Thirty-six (73%) of the patients in whom it was recommended received the treatment with five receiving the treatment by another care team. We reviewed the response to IVIG for patients under our care.

### Intravenous immunoglobulin treatment

Table 1 outlines the starting and current (or ending) dosing for all patients. The most common dose initiated was 2 g/kg monthly with the most common dosing 1 g/kg/day for 2 days monthly. Of the 31 patients, the dose and/or frequency of treatment was adjusted in 13 (42%) to provide an optimal effect. The dose may have been decreased to minimize adverse effects, whereas the treatment interval may have been increased or decreased if the effect appeared to last longer or shorter than the original 4 week interval. Overall, 10% and 40% of the patients, respectively, did best on a higher and lower dose than 2 g/kg/month.

**Table 1:** Starting and Most Recent Dosing Schedule for Intravenous Immunoglobulin (IVIG) Treatment

Starting dosing schedule	Monthly dose	Number of patients	Most recent dosing schedule^a^	Monthly dose	Number of patients
2.0 g/kg × 1 days monthly	2.0 g/kg	10% (3/31)	1.0 g/kg × 2 days every 3 wks	2.7 g/kg	6% (2/30)
1.0 g/kg × 2 days monthly	2.0 g/kg	74% (23/31)	0.8 g/kg × 3 days monthly	2.4 g/kg	3% (1/30)
0.75 g/kg × 2 days monthly	1.5 g/kg	3% (1/31)	2.0 g/kg × 1 days monthly	2.0 g/kg	10% (3/30)
1.0 g/kg × 1 days monthly	1.0 g/kg	3% (1/31)	1.0 g/kg × 2 days monthly	2.0 g/kg	37% (11/30)
0.8 g/kg × 1 days monthly	0.8 g/kg	7% (2/31)	1.0 g/kg × 1 days every 2 wks	2.0 g/kg	3% (1/30)
1.0 g/kg × 2 days once		3% (1/31)	0.8 g/kg × 1 days every 2 wks	1.6 g/kg	3% (1/30)
			0.75 g/kg × 2 days monthly	1.5 g/kg	3% (1/30)
			2.0 g/kg × 1 day every	1.3 g/kg	3% (1/30)
			1.0 g/kg × 1 day every 3 wks	1.3 g/kg	10% (3/30)
			1.3 g/kg × 1 day monthly	1.3 g/kg	3% (1/30)
			1.0 g/kg × 1 day monthly	1.0 g/kg	3% (1/30)
			0.8 g/kg × 1 day monthly	0.8 g/kg	6% (2/30)
			1.0 g/kg × 1 day every 6 wks	0.7 g/kg	3% (1/30)
			0.4 g/kg × 1 day monthly	0.4 g/kg	3% (1/30)

^a^Only the 31 patients that received IVIG under the care of the authors are included in this section

All patients started on monthly dosing but 30% changed to a different dosing interval, with 17%, 6%, and 6% receiving the treatment at 3, 6, and 2 week intervals, respectively. In several cases, extending the treatment interval resulted in behavioral regression, requiring shortening the interval to restore treatment effectiveness (See Case #3 below).

### Parent-reported outcomes

The majority of the parents reported improvements in communication and/or language with fewer reporting improvements in aberrant behavior (Table 2). About a quarter reported improvement in repetitive behavior and academics. Between 10% and 30% reported improvements in social interactions, tics, motor function, and seizures, only three parents did not report improvements. Seventy-one percent (22/31) of patients reported improvements in two or more symptoms and 90% (28/31) of the patients demonstrated improvements in one or more symptoms (Table 3).

**Table 2:** Parental Reported Symptomatic Improvements with Intravenous Immunoglobulin (IVIG) Treatment

Symptoms improvement	Patients reporting improvement
Communication & language	58% (18/31)
Aberrant behavior	35% (11/31)
Repetitive behavior	23% (7/31)
Academics	23% (7/31)
Social interactions	23% (7/31)
Tics	16% (5/31)
Motor	16% (5/31)
Other	16% (5/31)
Seizures	10% (3/31)
None	10% (3/31)

**Table 3:** Number of Symptoms Reported Improved with Intravenous Immunoglobulin (IVIG) Treatment

Number of patients	Number of symptoms improved
10% (3/31)	0
19% (6/31)	1
35% (11/31)	2
13% (4/31)	3
13% (4/31)	4
6% (2/31)	5
3% (1/31)	6

### Behavioral questionnaire outcomes

For 21 and 20 of the treated children the SRS and ABC behavioral questionnaires were acquired both before and during IVIG treatment, respectively.

**Fig. 2:**
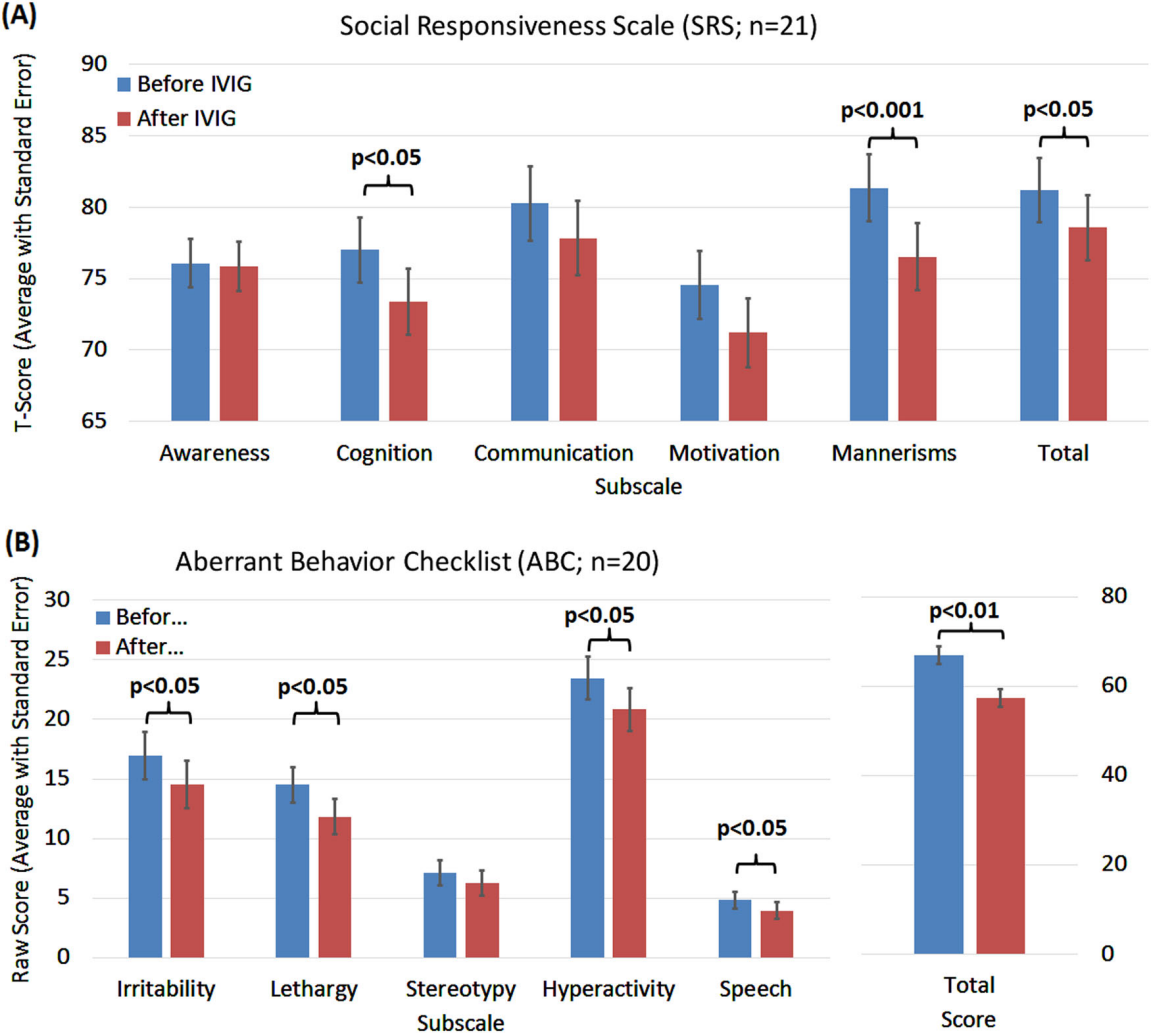
Changes in the Social Responsiveness Scale (SRS) and Aberrant Behavior Checklist (ABC) with intravenous immunoglobulin (IVIG) treatment

In Fig. 2a, all subscales of the SRS demonstrated improvement with IVIG treatment, although only a few of the improvements reached statistical significance (Fig. 2a). SRS subscales for cognition (F(1,57) = 4.71, *p* < 0.05, *d’* = 0.58) and mannerisms (F(1,57) = 13.34, *p* < 0.001, *d’* = 0.97) significantly improved with medium and large effect sizes, respectively. The total SRS score improved significantly with a medium effect size (F(1,57) = 4.40, *p* = 0.04, *d’* = 0.57). Change in communication (F(1,57) = 2.60, *p* = 0.11, *d’* = 0.43) and motivation (F(1,57) = 2.99, *p* = 0.09, *d’* = 0.46) SRS subscales were borderline significant with medium effect sizes, whereas the change in the awareness subscale was not statistically significant (F(1,57) = 0.02, *p* = 0.89, *d’* = 0.04).

In Fig. 2b, all subscales of the ABC demonstrated improvement with IVIG treatment with all but one reaching statistical significance (Fig. 2b). ABC subscales for irritability (F(1,57) = 5.81, *p* = 0.02, *d’* = 0.64), lethargy (social withdrawal) (F(1,57) = 5.75, *p* = 0.02, *d’* = 0.64), hyperactivity (F(1,57) = 4.63, p = 0.04, *d’* = 0.57) and inappropriate speech (F(1,57) = 5.13, *p* = 0.02, *d’* = 0.60) significantly improved with medium effect sizes. The total ABC score improved significantly with a large effect size (F(1,57) = 9.88, *p* = 0.003, *d’* = 0.83). The improvement in the ABC stereotypy subscales was not significant and had a small effect size (F(1,57) = 1.90, *p* = 0.17, *d’* = 0.36).

### Relationship of treatment response to immune biomarkers

**Fig. 3:**
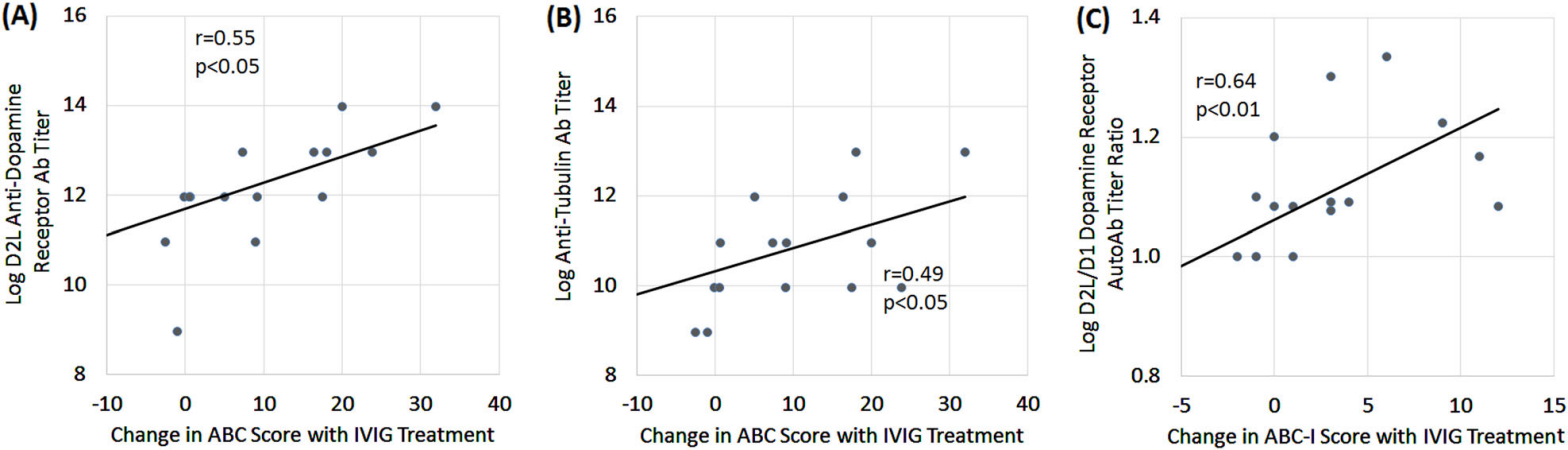
Baseline antibody titers are related to improvement in Aberrant Behavior Checklist (ABC) scores following intravenous immunoglobulin (IVIG). Greater improvement in ABC was related to high titers of antidopamine D2 receptor (D2L) antidopamine and anti-tubulin autoantibodies. A higher ratio of D2L to D1 antidopamine antibody titer was related to greater improvements in the irritability subscale of the ABC with IVIG treatment

Biomarkers and behavioral questionnaires were available for 17 children for the SRS and 16 children for the ABC. Of the five biomarkers, the change in the total ABC score resulting from IVIG treatment was associated with the change in the anti-D2R antibody (*r* = 0.55, *p* < 0.05; Fig. 3a) and anti-tublin Antibody (*r* = 0.49, *p* < 0.05; Fig. 3b). In addition, as hypothesized, the anti-D2L titer/anti-D1 titer ratio was significantly associated with the Irritability subscale of the ABC (*r* = 0.64, *p* < 0.01; Fig. 3c).

### IVIG responder analysis

**Fig. 4:**
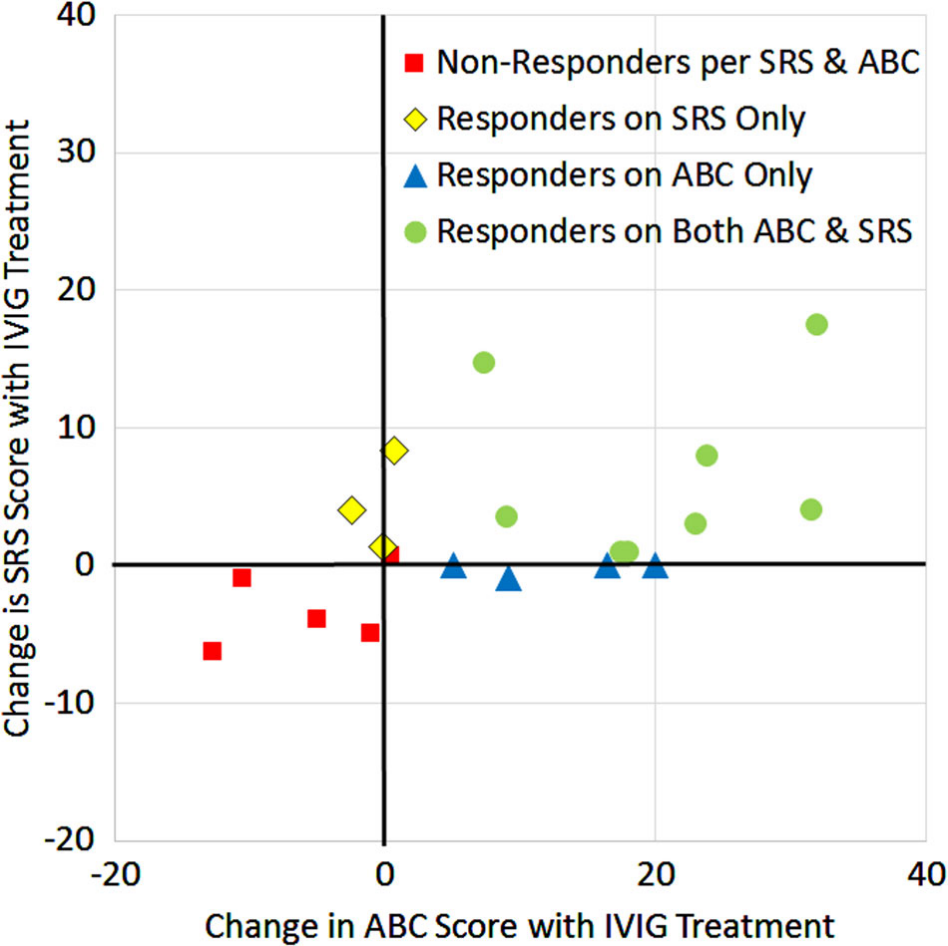
Responders to intravenous immunoglobulin (IVIG) was defined by the Social Responsiveness Scale (SRS) and Aberrant Behavior Checklist (ABC). Red squares are non-responders based on either questionnaire. Yellow diamonds were responders only based on the SRS but not the ABC. Blue triangles were responders based on the ABC but not the SRS. Green circles are responders based on both the ABC and SRS

The change in SRS scores is modest when it occurs, whereas the change in ABC scores can be rather marked when it occurs (Fig. 4). Change in scores of the two questionnaires correlated well with each other (*r* = 0.52, *p* = 0.01). Based on the distribution of scores a cutoff ≥ + 1 used to define a responder for the questionnaire scores. Using this criterion, five (25%) individuals were IVIG non-responders based on both questionnaires, three (15%) were IVIG responders based on the SRS only, four (20%) were IVIG responders based on the ABC only, and eight (40%) were responders based on both questionnaires. A criterion of two or more reported improvements in symptoms was used as a cutoff for parental reported improvement. Using these criteria, 52%, 60%, and 65% of patients were responders as defined by the SRS, ABC, and parental report. Thus, we believe that the criterion we used separated the groups well into responders and non-responders.

The Cunningham panel predicted response to IVIG treatment with an accuracy of 81% with a sensitivity of 90% and a specificity of 67% based on the ABC scores; with an accuracy of 88% with a sensitivity of 100% and a specificity of 75% based on the SRS scores; and with an accuracy of 88% with a sensitivity of 100% and a specificity of 67% based on parental scores.

### Temporal characteristic of response

**Fig. 5:**
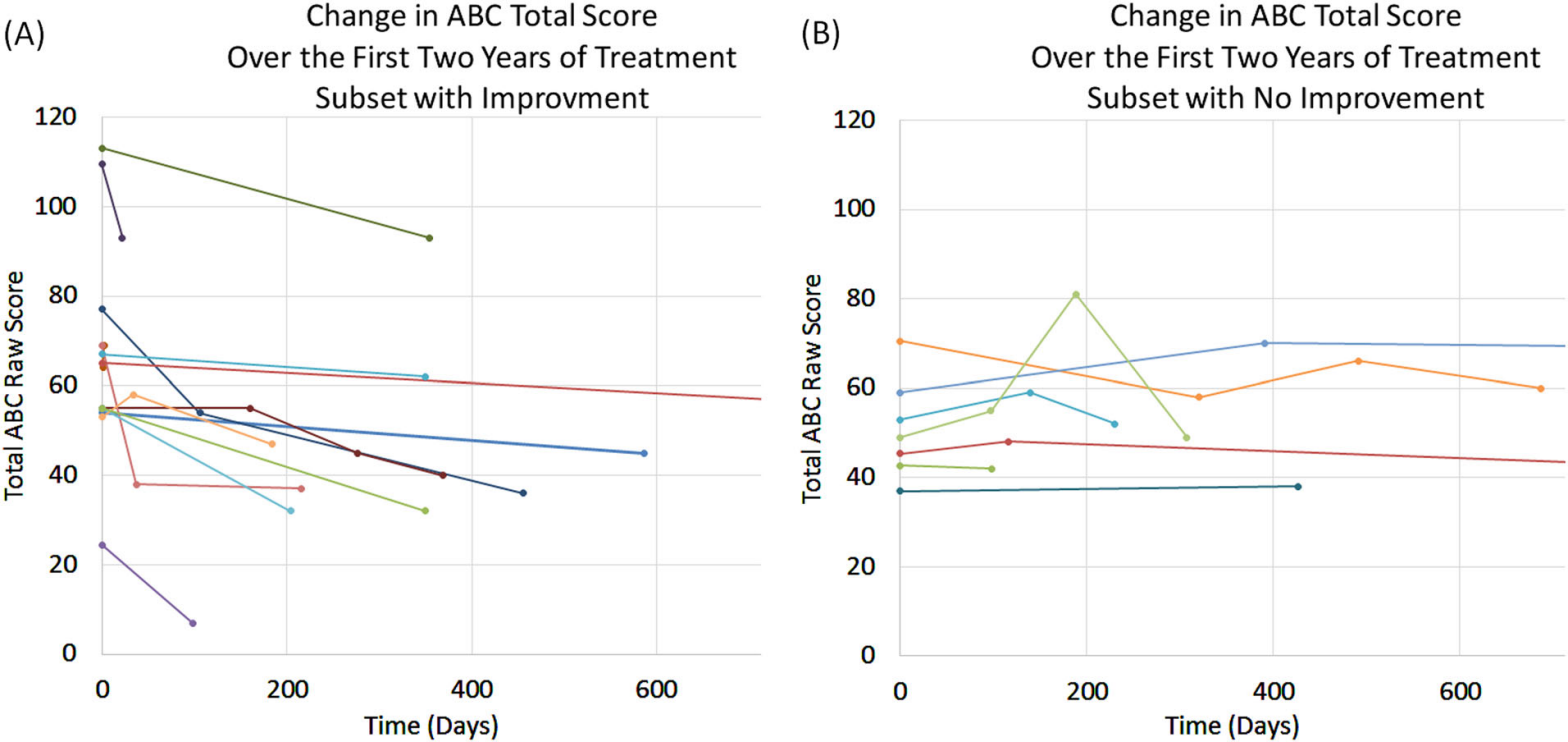
Change in the total Aberrant Behavior Checklist (ABC) score over the first two years of treatment as compared to the average total ABC score prior to treatment. The figure is divided up into (**a**) treatment responders and (**b**) treatment non-responders, as defined by the ABC

The speed at which behavioral effects of the treatment arose and the duration of these effects were rather variable from patient to patient. However, ABC responders (Fig. 5a) demonstrated a clear response that was variable in the rate at which it improved across patients, with some patients showing a very rapid decrease in ABC score and others showing a gradual improvement in ABC score. In contrast, non-responders showed a variable response with ABC score worsening and improving over time for many. One non-responder demonstrated a bout of severe hyperactivity that transiently increase the total ABC score dramatically.

### Long-term outcomes

Of the 31 patients who were initially treated with IVIG, 77% (24/31) continued with IVIG treatment long-term (> 1 year). The majority of these 24 patients, 88% (21/24), found continuing benefit from the treatment. Of the seven that stopped the IVIG treatment, 29% (2/7) stopped because of lack of insurance coverage, 29% (2/7) stopped because adverse effects, 14% (1/7) stopped in order to try a more intense immunosuppression treatment, 14% (1/7) stopped as a trial and requested to restart because of behavioral regression and 14% (1/7) was only prescribed one treatment. Only 6% (2/31) of the patients stopped the treatment because of lack of benefit and only 10% (3/13) who did not stop the treatment found limited continuing benefit.

### Adverse effects

Sixty-five percent (20/31) of patients reported adverse effects, most commonly headaches and vomiting (Table 4). Many adverse effects were limited to the 48–72 h period immediately following the infusion. Treatments were individualized to each patient to minimize adverse effects by prescribing pre-infusion anti-pyretic (ibuprofen 10 mg/kg oral; PO), steroids (solumedrol 1 mg/kg intravaneous; IV), fluids (~ 20 ml/kg ½ normal saline over 1 h), anti-emetics (ondansetron 0.15 mg/kg IV), anti-histamine (diphenhydramine 0.5 mg/kg IV/PO) and/or post infusion fluids. These pretreatments were prescribed in 84%, 81%, 32%, 26%, 19%, and 6%, respectively. Three patients were prescribed anti-emetics up to 72 h post infusion. One patient with baseline idiopathic hypertension was given aggressive hydration pre and post infusion to prevent fluctuation in blood pressure.

**Table 4:** Adverse Effects Associated with Intravenous Immunoglobulin (IVIG) Treatment

Adverse effect	Patients reporting
Headache	39% (12/31)
Vomiting	29% (9/31)
Worsening behavior	16% (5/31)
Anxiety	13% (4/31)
Fever	13% (4/31)
Nausea	10% (3/31)
Fatigue	10% (3/31)
Rash	6% (2/31)

### Selected example cases

#### Case 1

**Fig. 6:**
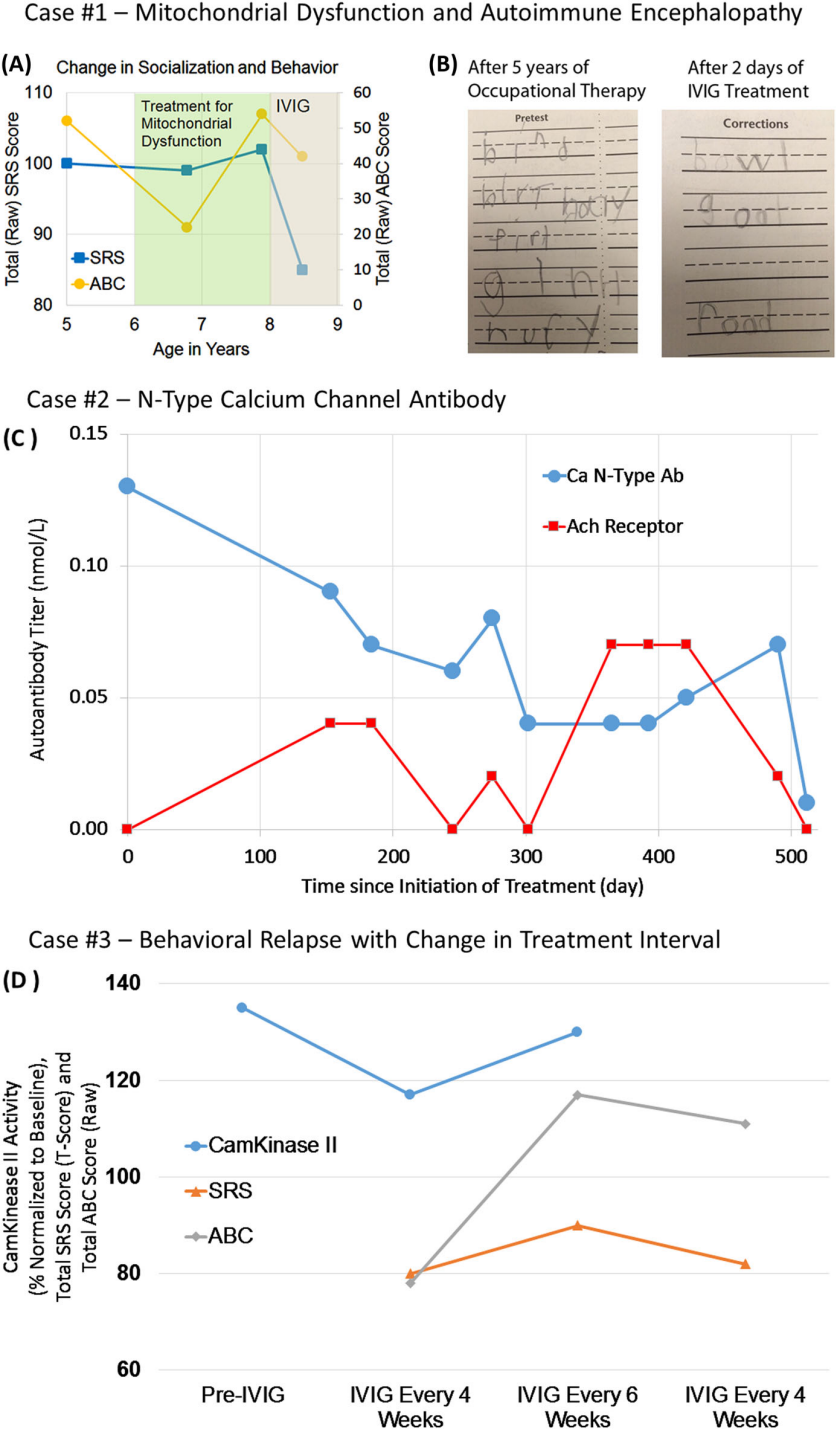
Case examples of responders to intravenous immunoglobulin (IVIG) treatment. Case #1 is a child with autism spectrum disorder (ASD) and mitochondrial dysfunction that had a sudden worsening in behavior at 8 years of age. **a** Initiating IVIG significantly improved behavior as measured by the Aberrant Behavior Checklist (ABC) and social function as measured by the Social Responsiveness Scale (SRS). **b** The writing sample on the left shows the patient’s handwriting a few months before IVIG treatment, and the sample on the right shows the patient’s handwriting 2 days after his first IVIG treatment. Case #2 is a boy with sudden regression found to have an N-Type Voltage-Gated Calcium-Channel autoantibody. **c** IVIG treatment significantly improved autoantibody titers. Case #3 is a girl with multiple regressions and severe ASD and related symptoms. **d** IVIG normalized CamKinase II activity but CamKinase II activity increased to abnormal levels and behavior, as measured by the ABC and SRS, worsened with increasing the IVIG treatment interval to every 6 weeks. Behavior improved after reinstating the original every 4 weeks dosing interval

At 15 months of age, a male patient experienced sudden regression in speech, eye contact, and fine motor skills. After 6 years of speech and occupational therapy, he could speak 3–4 word sentences but was not able to engage in reciprocal conversation, exhibited limited eye contact, and suffered from severe restricted interests, anxiety, and frequent meltdowns. A medical evaluation demonstrated that he had biomarkers consistent with mitochondrial dysfunction, as manifested by repeated elevated lactate, pyruvate, and alanine-to-lysine ratio (mean (*N*,SD) 2.3 mM/L (25,0.65), nl < 1.3; 0.20 mM/L (24,0.5), nl < 0.08; 4.0 (26,1.96), nl < 2.5, respectively) and mitochondrial buccal swab demonstrating depressed citrate synthase (55%) and elevated complex I (178%) and IV (236%) function. A mitochondrial cocktail was started, including ubiquinol, l-carnitine, creatine monophosphate, niacin, pantothenic acid, pyridoxine, riboflavin, thiamine, cobalamin, and folinic acid. During the first treatment year he demonstrated improved adaptability, socialization, and ASD-related behavior. However, improvements in socialization plateaued and behavior worsened (See Fig. 6a), prompting further investigation. A Cunningham panel showed elevations in anti-tubulin antibodies (2000; nl = 250–1000) and CaMKII (151, nl < 130), prompting a trial of IVIG at 1 g/kg/day × 2 days every month. Within 2 days of the IVIG treatment, his handwriting significantly improved (See Fig. 6b). With each subsequent treatment he progressively gained skills, including significant improvements in language. After 5 months, he began having reciprocal conversations and was able to describe how IVIG made him “feel”. After 6 months, his restricted interests resolved. After 18 months of IVIG treatment, he has been able to sit with peers in a noisy gymnasium and engage in social conversation as part of a small group.

#### Case 2

At ~ 3½ years of age, a previously normally developing child underwent a slow regression lasting 6 months. His parents pursued a language assessment at the beginning of the regression because he became less verbal but the evaluation did not demonstrate any significant abnormalities. However, as the third year of his life progressed, he lost speech, social interactions, developed repetitive movements, and sleep disturbance with an inability to stay asleep throughout the night. A repeat language evaluation at 3 years 11 months demonstrated a significant (17 standardized point) loss in language ability as compared with the evaluation earlier in the year. At 4 years of age he was diagnosed with ASD. Gluten- and dairy-free diet reportedly improved focus and attention and speech, occupational, physical, and applied behavioral analysis therapies began. Family history was remarkable for learning disabilities in maternal family and developmental delays in the paternal family, but the mother, father, and sibling did not have a history of neurodevelopmental disorders. He was otherwise healthy.

He was evaluated in our clinic at 4 years 6 months of age. On examination, he was active, cooperative, and anxious with very limited verbal communication skills but demonstrated eye contact. A mitochondrial and genetic workup (Chromosomal Microarray and Fragile X) and overnight EEG were unremarkable. FRA, NMDA, and GAD65 antibodies were negative, however, PNP demonstrated an elevated N-Type Calcium-Channel antibody (0.13 nmol/L; nl < 0.03 nmol/L). A magnetic resonance imaging scan of the brain was normal and lumbar puncture demonstrated low protein (16; nl ≥ 20).

IVIG treatment was initiated at 1 g/kg/day for 2 days. Four weeks after the first IVIG infusion a marked improvement occurred in near constant chewing, repetitive movement, and severe sleep disruption. Five months after the first treatment, some ASD symptoms started to return, resulting in starting IVIG treatments on a monthly to every other month schedule. The N-type VGCC antibody dropped to 0.09 nmol/l just prior to the second IVIG treatment and then progressively further with additional treatments, reaching a low of 0.4 nmol/l for several months, and eventually dropping to 0.01 nmol/L (Fig. 6c). The ABC total score dropped from 69 before treatment to 38 just before the second treatment with this decrease paralleling the decrease in the N-type VGCC antibody. Interestingly, there was an increase in the neuronal acetylcholine receptor autoantibody during the treatments course, which eventually decreased along with the N-type VGCC antibody.

#### Case 3

This girl developed normally until about 3 years of age when she experienced periodic episodes of abrupt loss of previously acquired skills and developed other neuropsychiatric symptoms. The most significant of these regressions was at 4 years of age when she developed an abrupt onset of repetitive behaviors, tics, poor concentration, emotional liability, separation anxiety, urinary frequency and urgency, sleep disturbance, and aggressive behavior. She was diagnosed with ASD at age 8 years of age. At 9 years of age she developed partial complex seizures, incompletely controlled by lamotrigine. At 7 years of age, a Cunningham panel demonstrated a CaMKII (135; nl < 130; See Fig. 6d) elevation. She was treated with IVIG 1 g/kg/day for 2 days every month. Improvements occurred in tics, academics, and seizure frequency, and CaMKII normalized (Fig. 6d). After 6 months of treatment, the interval of IVIG was extend to every 6 weeks. The child’s behavior quickly regressed with a significant increase in both the SRS and ABC scales and an increase in the CaMKII in the abnormal range again (Fig. 6d). After 6 months on monthly IVIG, her behavior improved (Fig. 6d).

## Discussion

In this study, we examined 82 children with neurodevelopmental disorders for biomarkers consistent with AIE and considered for immunomodulatory treatment for those with a clinical presentation and laboratory values consistent with AIE. Of those who underwent IVIG treatment using our care team, the majority had symptoms improvements as measured by standardized questionnaires. Although adverse effects were reported for the majority of patients, they were mostly limited to the time around the IVIG infusion, resulting in the great majority of patients considering the benefits outweighing any adverse effects.

Interestingly, in our cohort of patients who presented to our ASD clinic, very few demonstrated autoantibodies usually associated with AIE in children. Interestingly, none of the patients was positive for NMDA receptor or VGKC autoantibodies. Only 5% of the patient cohort was positive for the GAD65 autoantibody with only one GAD65 titer within a clinically significant range. This low rate of positivity could explain the contradictory reports of the association of the GAD65 autoantibody with ASD as the rate of positivity may be very dependent on the sample of ASD children studied^40,60^. The child with ASD and the highest GAD65 titer also had type 1 diabetes, which is a disorder known to be associated with the GAD65 autoantibody^31^. This child was a responder to IVIG. Interestingly, GAD65 may be functionally disrupted in ASD in other ways besides an autoantibody mechanism. For example, GAD65 is downregulated in the prenatal valproic acid and MIA animal models of ASD^61–65^ and in post-mortem brain from individuals with ASD^66,67^, suggesting that it is a potentially important target of pathology in individuals with ASD.

This is the first reported association of VGCC autoantibodies with ASD. The VGCC is associated with a wide range of cognitive and motor disturbances^68^ and cerebellar degeneration in childhood^69^. The child positive for the N-type VGCC demonstrated an abrupt onset of sleep disturbance, repetitive behavior, and chewing on objects in addition to language and social regression at the onset of this disorder. IVIG treatment significantly and quickly improved these symptoms. Interestingly, like the GAD enzyme, ASD has been associated with non-autoimmune disruption in calcium-channel function^70^. Thus, some autoantibodies in individuals with ASD may merely represent one mechanism that can disrupt common pathophysiological disturbances leading to ASD.

Overall, the majority of parents of the patients treated with IVIG found the treatment beneficial and believed that the benefits outweighed any adverse effects. Close observation was important to tailor the dose and frequency of the treatment and minimize adverse effects. Consistent with previous literature, IVIG treatment in this study improved scores on the ABC^41,44^ and social interactions^42^. Of the six studies using IVIG in the treatment of ASD, three open-label studies, totaling 63 patients^41–43^ and one double-blind placebo-controlled study totaling 12 patients^44^ demonstrated a benefit, whereas two smaller open-label studies, totaling 15 patients^45,46^, did not show benefit. Two of the studies that demonstrated benefit selected individuals with immune system abnormalities, like the current study^42,43^. In contrast, the two open-label studies that did not demonstrate benefit did not select for immune abnormalities^45,46^. In addition, all previous studies used a lower dose of IVIG, usually 0.4 g/kg monthly. Some have observed better response with higher doses of IVIG^71^ potentially explaining the high rate of response in our study. Clearly more research is needed to better understand which subset of children with ASD can best benefit from IVIG as well as the optimal dose and interval for the treatment.

The majority of the ASD patients who had autoantibodies demonstrated elevations in autoantibodies measured by the Cunningham panel along with an elevation in the activation of CaMKII. Our analysis suggests that two of the autoantibodies, the anti-tubulin and anti-D2R were associated with responsiveness to IVIG treatment, suggesting that these could be biomarkers to select individuals who might benefit most from IVIG treatment. It should be noted that the participants were selected based on an elevated CaMKII, so this biomarker may have been artificially fixed with a limited dynamic range because of our patient selection process, thereby decreasing its ability to be a predictive biomarker. As the ABC was most closely associated with immune biomarkers, it may be that the autoantibodies identified as predictive of response to IVIG may be closely associated with modulation of behavior as has been suggested in Sydenham chorea^22^ and PANDAS^23,26^.

The limitations of this study include the small sample size and the questionnaire-based outcome measures. Furthermore, this study was not a systematic clinical trial with comprehensive measurements of behavior, cognition, and language. Thus, further studies are needed to better identify which specific symptoms are targeted by IVIG treatment as well as the most-sensitive biomarkers to identify which patients are most likely to respond to IVIG treatment. Despite these limitations, our study verifies previous reports that suggest that IVIG may be useful in individuals with ASD who demonstrate biomarkers of immune abnormalities.

## Electronic supplementary material

Table S1
