# Supplementary material for: Intravenous immunoglobulin for the treatment of autoimmune encephalopathy in children with autism
Source: Transl Psychiatry. 2018 Aug 10;8:148. doi: 10.1038/s41398-018-0214-7 (PMC6086890; doi:10.1038/s41398-018-0214-7)
Supplement: Supplementary file 1 — Table S1 [file 41398_2018_214_MOESM1_ESM.docx]

Table S1. Demographic and Clinical Characteristics at time of initiating immune workup

| **Variable** | **All Evaluated**  **(n=82)** | **IVIG under Team**  **(n=31)** |
| --- | --- | --- |
| Age, years months, mean (standard deviation) | 9y 10m (4y 1m) | 9y 9m (4y 5m) |
| Males, N (%) | 58 (71%) | 19 (61%) |
| **Ethnicity/Race, N (%)** |  |  |
| Non-Hispanic Caucasian | 67 (82%) | 27 (87%) |
| Hispanic | 6 (7%) | 2 (6%) |
| Non-Hispanic Asian | 5 (6%) | 1 (3%) |
| Non-Hispanic Mixed Race | 4 (5%) | 1 (3%) |
| Non-Hispanic African American | 0 (0%) | 0 (0%) |
| **State of Residence, N (%)** |  |  |
| Arkansas | 21 (26%) | 13 (42%) |
| Out of State | 58 (71%) | 18 (58%) |
| Out of Country | 3 (4%) | 0 (0%) |
| Autism Spectrum Disorder (ASD) Diagnosis, N (%) | 80 (98%) | 29 (94%) |
| **ASD Diagnostic Documentation, N (%)** |  |  |
| Autism Diagnostic Observation Schedule | 17 (21%) | 6 (19%) |
| Autism Diagnostic Interview-Revised | 12 (15%) | 4 (13%) |
| 3 Practitioner Agreement | 35 (43%) | 13 (42%) |
| Single Practitioner with standardized questionnaires | 26 (44%) | 14 (44%) |
| Outside Report of Diagnosis | 8 (10%) | 3 (10%) |
| **Cunningham Panel, N (%)** |  |  |
| Positive | 44 (54%) | 19 (61%) |
| Negative | 33 (40%) | 8 (26%) |
| Not Assessed | 5 (6%) | 4 (13%) |
| **Paraneoplastic Panel*, N (%)** |  |  |
| Positive | 4 (5%) | 1 (3%) |
| Negative | 59 (72%) | 20 (65%) |
| Not Assessed | 19 (23%) | 10 (32%) |
| **Glutamic Acid Decarboxylase (GAD65) antibody*, N (%)** |  |  |
| Positive | 3 (4%) | 3 (10%) |
| Negative | 57 (70%) | 16 (52%) |
| Not Assessed | 21 (26%) | 12 (39%) |
| **Complement C3*, N (%)** |  |  |
| Positive | 0 (0%) | 0 (0%) |
| Negative | 18 (22%) | 13 (42%) |
| Not Assessed | 64 (78%) | 18 (58%) |
| **Complement C4*, N (%)** |  |  |
| Positive | 1 (1%) | 1 (3%) |
| Negative | 17 (21%) | 11 (35%) |
| Not Assessed | 64 (78%) | 19 (61%) |
| **Complement, Total (CH50)*, N (%)** |  |  |
| Positive | 3 (4%) | 1 (3%) |
| Negative | 15 (18%) | 11 (35%) |
| Not Assessed | 64 (78%) | 19 (61%) |
| **C Reactive Protein*, N (%)** |  |  |
| Positive | 1 (1%) | 1 (3%) |
| Negative | 23 (28%) | 8 (26%) |
| Not Assessed | 58 (71%) | 22 (71%) |
| **Erythrocyte Sedimentation Rate (ESR)*, N (%)** |  |  |
| Positive | 4 (5%) | 3 (10%) |
| Negative | 16 (20%) | 5 (16%) |
| Not Assessed | 62 (76%) | 23 (74%) |
| **Folate-Receptor Autoantibody, N (%)** |  |  |
| Positive-Binding | 4 (5%) | 1 (3%) |
| Positive-Blocking | 10 (12%) | 3 (10%) |
| Positive-Binding or Blocking | 11 (13%) | 3 (10%) |
| Negative | 6 (7%) | 1 (3%) |
| Not Assessed | 65 (79%) | 27 (87%) |
| **N-Methyl-D-Aspartate Receptor autoantibody *, N (%)** |  |  |
| Positive | 0 (0%) | 0 (0%) |
| Negative | 34 (41%) | 12 (39%) |
| Not Assessed | 48 (59%) | 19 (61%) |
| **Antistreptolysin O (ASO) titer*, N (%)** |  |  |
| Positive | 7 (9%) | 2 (6%) |
| Negative | 16 (20%) | 7 (23%) |
| Not Assessed | 59 (72%) | 22 (71%) |
| **Anti-Deoxyribonuclease B titer*, N (%)** |  |  |
| Positive | 5 (6%) | 3 (10%) |
| Negative | 9 (11%) | 3 (10%) |
| Not Assessed | 68 (83%) | 25 (81%) |
| **Medications, N (%)** |  |  |
| Gastrointestinal Medications | 55 (67%) | 19 (61%) |
| Antimicrobial Medications | 32 (39%) | 13 (42%) |
| Allergy/Asthma Medications | 27 (33%) | 14 (45%) |
| Melatonin | 22 (27%) | 10 (32%) |
| Other Psychotropic Medications | 20 (24%) | 11 (35%) |
| Antiepileptic Medication | 20 (24%) | 8 (26%) |
| Immunomodulatory/Anti-inflammatory Medications | 18 (22%) | 7 (23%) |
| Alpha-adrenergic agonists | 12 (15%) | 6 (19%) |
| Selective Serotonin Reuptake Inhibitors | 12 (15%) | 6 (19%) |
| Beta Blocker | 8 (10%) | 5 (16%) |
| Antipsychotic | 7 (9%) | 3 (10%) |
| Stimulant | 6 (7%) | 4 (13%) |
| Diuretic | 3 (4%) | 3 (10%) |
| Anticholinergic | 3 (4%) | 0 (0%) |
| Beta-Adrenergic | 1 (1%) | 1 (3%) |
| Chemotherapy | 1 (1%) | 0 (0%) |
| GABA Agonist | 1 (1%) | 0 (0%) |
| **Supplements, N (%)** |  |  |
| Minerals | 49 (60%) | 20 (65%) |
| Other Vitamins (Not B Vitamins) | 47 (57%) | 16 (52%) |
| Folate | 40 (49%) | 14 (45%) |
| Carnitine | 39 (48%) | 17 (55%) |
| Fatty Acids | 35 (43%) | 12 (39%) |
| Vitamin B-12 | 33 (40%) | 12 (39%) |
| Other Antioxidants | 32 (39%) | 7 (23%) |
| Amino Acids | 29 (35%) | 8 (26%) |
| Other B Vitamins (Not B-12) | 28 (24%) | 9 (29%) |
| CoEnzyme Q10 | 25 (30%) | 11 (35%) |
| Herbal Supplements | 19 (23%) | 7 (23%) |
| Multivitamin | 17 (21%) | 7 (23%) |
| Hormone | 9 (11%) | 6 (19%) |
| Thyroid Supplements | 8 (10%) | 3 (10%) |
| Dietary Supplement | 5 (6%) | 2 (6%) |
| **Comorbid Medical Conditions per Parent Report, N (%)** |  |  |
| Gastrointestinal Symptoms | 70 (85%) | 27 (87%) |
| General Health Symptoms | 64 (78%) | 24 (77%) |
| Neurological Symptoms | 58 (71%) | 20 (65%) |
| Allergic/Asthma Symptoms | 53 (65%) | 22 (71%) |
| Immune/Metabolic Abnormalities | 52 (63%) | 21 (68%) |
| Psychiatric Symptoms | 43 (52%) | 19 (61%) |
| Growth Abnormalities | 26 (32%) | 12 (39%) |
| Genitourinary Symptoms | 15 (18%) | 9 (29%) |
| Endocrine Symptoms | 9 (11%) | 3 (10%) |
| Phelan-McDermid Syndrome | 2 (2%) | 1 (3%) |
| Cardiovascular Symptoms | 1 (1%) | 0 (0%) |
| **History of Regression, N (%)** |  |  |
| No History of Regression | 28 (73%) | 9 (29%) |
| History of Regression | 54 (67%) | 24 (71%) |
| Single Regression | 17 (21%) | 6 (19%) |
| Multiple Regressions | 37 (45%) | 16 (52%) |

*These are all the initial test results, in some these tests were completed more than once.
